# HPV genotypes in high-grade cervical lesions and invasive cervical carcinoma detected in Gabonese women

**DOI:** 10.1186/s13027-023-00493-z

**Published:** 2023-03-08

**Authors:** Pamela Moussavou-Boundzanga, Ismaël Hervé Koumakpayi, Corinne Engohan Aloghe, Junie K. Chansi, Rose Revignet, Eric M. Leroy, Nicolas Berthet

**Affiliations:** 1 Centre International de Recherches Médicales de Franceville (CIRMF), BP 69 Franceville, Gabon; 2 Laboratoire de Biologie Moléculaire et Cellulaire (LABMC), Université des Sciences et Techniques de Masuku, BP 941 Franceville, Gabon; 3 Centre Hospitalier Universitaire de Libreville, Libreville, Gabon; 4 Institut de Cancérologie de Libreville (ICL), Libreville, Gabon; 5 Institut de Recherches et de Développement (IRD), MIVEGEC, CNRS, IRD, Université de Montpellier, Montpellier, France; 6 Unité Environnement et Risques Infectieux, Cellule d’Intervention Biologique d’Urgence, Institut Pasteur, Université Paris-Cité, 25 Rue du Docteur Roux, 75724 Paris, France; 7 Unit of Discovery and Molecular Characterization of Pathogens, The Center for Microbes, Development and Health, Institut Pasteur of Shanghai - Chinese Academy of Sciences, Life Science Research Building, No 320 Yueyang Road, XuHui District, Shanghai, China

**Keywords:** High-grade cervical lesions, Invasive cervical cancer, HPV, Gabon

## Abstract

**Background:**

Cervical cancer is the third most common cancer among women worldwide, but particularly affects women living in sub-Saharan Africa. Screening and vaccination programs are two prevention approaches that can reduce cervical cancer incidence. However, effective vaccination campaigns require better knowledge of the prevalence of the main human papillomavirus (HPV) genotypes reported in high-grade neoplastic lesions and invasive carcinomas in women.

**Methods:**

All samples collected in this study were processed using standard histopathological methods with haematoxylin and eosin staining of the sections. Areas with abnormal cells were then identified. The HPV genotype was determined on the DNA extracted from the same sections using nested PCR followed by amplicon sequencing and real-time PCR specific to five different HPV genotypes (16, 18, 33, 45 and 58).

**Results:**

A total of 132 Gabonese patients with high-grade neoplastic lesions were included in this study; 81% were squamous cell carcinomas (SCC). At least one HPV was detected in 92.4% patients; HPV16 (75.4%) was the most frequent genotype, followed by HPV18, 58, 45, 33 and 35. Moreover, histological analysis showed that SCC samples had 50% and 58.2% stage III and IV tumor cells, respectively, according to the FIGO classification. Finally, 36.9% of these stage III and IV patients were less than 50 years old.

**Conclusions:**

Our results confirm the high prevalence of HPV16 and 18 genotypes among high-grade lesions in Gabonese women. This study confirms the need for a national strategy for early screening of precancerous lesions associated with a broad national vaccination program among non-sexually active women to significantly reduce the long-term cancer burden.

**Supplementary Information:**

The online version contains supplementary material available at 10.1186/s13027-023-00493-z.

## Introduction

Cervical cancer is a major global public health issue. Of the estimated 570,000 new cases and 311,365 deaths in 2018, it is the third most common cancer among women worldwide, but particularly affects women living in developing countries [1]. Approximately 85% of new cancer cases are reported in low- and middle-income countries. The various screening programs, particularly those based on the Papanicolaou smear, have reduced the rate of cervical cancer in developed countries. However, the high rates of cervical cancer observed in developing countries result from inadequate screening and treatment of precancerous lesions.

To reduce this disparity, the WHO has set up a strategy to ensure that 90% of girls are fully vaccinated against human papillomavirus (HPV) by the age of 15 and that 70% of women are tested for HPV at 35 and 45 years of age [2]. Although vaccination and screening are two prevention approaches, vaccination is probably the most effective in the long term given its extremely high preventive efficacy [3–5]. However, vaccination campaigns mainly benefit younger generations of women and future generations; thus, several decades will pass before the health effects of vaccination are observed in developing countries. However, in the meantime, screening programs can reduce the burden of cervical cancer more rapidly and benefit both current and future generations of women. Thus, screening programs combined with vaccination are fundamental, because they are potentially cost-effective and can reduce cervical cancer incidence and mortality rates [6].

Given that epidemiological data on HPV in low-income countries, particularly in sub-Saharan Africa (SSA), are difficult to compare, an African consortium of five French-speaking African countries (Senegal, Ivory Coast, Cameroon, Gabon and Madagascar) was created under the aegis of the National Cancer Institute (INCa) in France. The consortium focuses on providing a working model to establish standardized research protocols for the identification of HPV genotypes associated with high-grade invasive neoplasia intraepithelial lesions in women living in the consortium countries [7]. In these participating countries, cervical cancer is a major public health issue, and is generally the most common cancer found in women. As in other Central African countries, cervical cancer is one of the leading cause of cancer mortality in Gabon and remains a priority for the authorities of this country for better management [8]. However, the main challenge is to determine the prevalence of the different HPV genotypes found in cervical cancer in Gabon. Previous studies in Gabon have shown that the distribution of genotypes varies according to the type of population studied, age group, histological status and methods used. Overall, these studies have revealed a predominance of the HPV16, 18 and 33 genotypes in cervical cancers [9–11].

The objective of this study was to determine the prevalence of major HPV genotypes in all reported cases of high-grade neoplastic lesions and invasive carcinomas at the *Centre Hospitalier Universitaire d'Angondjé* (CHUA) and the *Hôpital des Instructions des Armées Omar Bongo Ondimba* (HIAOBO)between 2012 and 2017 in Gabon. The clinical stage of these lesions was determined and virological investigations were performed using standardized protocols common to all other consortium countries.

## Methods

### Population

A multi-center, cross-sectional study was conducted in two hospitals in Libreville (Gabon), at the *CHUA* and at the HIA OBO. Between 2012 and 2017, all women diagnosed histologically with either cervical intraepithelial neoplasia 3 (CIN3), adenocarcinoma (ADC) or squamous cell carcinoma (SCC) were included in this study. The exclusion criteria for the study were as follows: (i) patients with a history of total uterine or cervical resection or (ii) patients with a history of chemotherapy and (iii) vaccinated patients. In addition, samples for which patient age was unavailable or not precisely known were excluded. To avoid duplication, the names and date of birth of each patient were checked by two different technicians. All laboratory manipulations were carried out at the Centre Interdisciplinaire de Recherches Médicales de Franceville (CIRMF) in Gabon.

### Histological analysis from formalin-fixed paraffin-embedded (FFPE) tissue

The samples were processed using standard histopathological methods and evaluated by a certificated pathologist. All patients were staged according to the International Federation of Gynecology and Obstetrics (FIGO) staging system 2009 [12]. The abnormal cell percentage was calculated by selecting the area where the lesion was most representative on the slide and counting at high magnification (× 400) out of 100 cells. Only these regions with abnormal tissue were used for DNA extraction.

### Sample preparation for molecular analysis

Depending on the size of the biopsy, five to seven sections of 4 µm were cut from each FFPE block and placed on a slide. The outer sections were used for histological analysis and the inner sections were used for molecular investigations. Histological sections were stained with hematoxylin and eosin (H&E) and areas including abnormal cells were identified.

### DNA extraction

Before extraction, abnormal tissue sections from the five inner slides (each 4-μm thick) were placed in a 1.5 ml Eppendorf tube. Precaution was taken to avoid cross-contamination, including changing cutting blades and changing gloves between each preparation. Then, each block was deparaffinized. To dissolve the paraffin, blocks were treated with toluene for 2 min, and then immersed in 100% alcohol for 2 min to remove the toluene. Then, total DNA was extracted using a QIAamp DNA FFPE Tissue Kit (Qiagen, Redwood City, CA, USA) according to the manufacturer’s instructions. Extracted DNA was stored at − 20 °C until analysis. DNA was quantified using a Qubit® dsDNA BR Assay kit with the Qubit 2.0 fluorimeter (Life Technologies, Carlsbad, CA, USA). DNA quality was assessed using real-time qPCR to amplify a 107 bp fragment of the glyceraldehyde-3-phosphate dehydrogenase (GADPH) housekeeping gene.

### Identification of HPV genotypes using nested PCR assay

The HPV genome in the DNA extracted from FFPE samples was genotyped using nested PCR followed by amplicon sequencing. The nested PCR used primers derived from the MY09/MY011 region of the L1 gene of the HPV genome and GP5 + /6 + consensus inner primers as described previously [9]. The amplification result was visualized on electrophoresis agarose gels stained with ethidium bromide and observed on a UV transilluminator. Results were deemed positive based on the presence of a 150 bp band and the sequenced amplicon corresponding to HPV upon a BLAST analysis. The amplicon was sequenced performed using the BigDye Terminator v3.1 cycle sequencing kit according to the manufacturer’s protocol.

### Identification of HPV genotypes using real-time PCR

Real-time PCR was carried out to detect five different HPV genotypes (16, 18, 33, 45 and 58) [13]. Each PCR reaction was performed in a 25 ul volume using 50 ng of template DNA in 5 ul, 12.5 ul of Platinum Quantitative PCR SuperMix-UDG (Invitrogen), 10 pmol of each primer and 5 pmol of each probe. Thermal cycling was based on the following program: an initial denaturation step at 95 °C for 10 min, 55 amplification cycles (denaturation at 95 °C for 15 s, annealing at 50 °C for 20 s and extension at 60 °C for 40 s each). A positive and a negative control were included in each amplification reaction.

### Determination of infectious profile

The result of any method was compiled independently. Specific HPV genotypes concurrently detected by both RealTime PCR and nested PCR were regarded as valid results. In the event of a discordant result, the result was grouped into High risk, Low risk, and uncertain-risk groups according to previously described criteria [14]. An individual sample was considered co-infections if at least two different genotypes were detected by either of the two methods.

### Statistical analysis

Statistical analysis were performed using R version 3.2.2. Summary statistics for age at histological diagnosis (n, mean, standard error, median, minimum and maximum) and cervical lesion (n; proportion) were produced. The associations between HPV infection and both age groupings and severity of histological diagnosis have been tested by chi-square at 95% confidence level.

## Results

### Characteristics of the study population

Among the 191 patients followed in the two laboratories between 2012 and 2017, only 132 were included in this study due to missing data for some patients. The population was aged between 24 and 87 years; the mean age of the cohort was 54.9 years (SD ± 12.8 years). The majority of women were postmenopausal 76.5% (101/132) and multiparous 84.6% (77/91) with a maximum of 12 pregnancies. Moreover, age at first sexual intercourse varied between 10 and 22 years (median = 16 years). Only 35.6% (36/101) had never been to school. Histological analyses showed that 6.82% (9/132) of the patients had ADC, 12.12% (16/132) had CIN3 and SCC was found in 81.06% (107/132) of included patients (Table 1). The mean age of patients with CIN3 was 46.7 years (SD ± 10.1 years), 55.1 years (SD ± 12.1 years) for ADC and 56.1 years (SD ± 12.8 years) for SCC. In addition, histological analyses showed that the percentage of tumor cells varied between 5 and 100% depending on the histological diagnosis of the lesions. For instance, more than 50% of SCCs had more than 50% tumor cells, but 62.5% of CIN3 cases had between 5 and 50% tumor cells (Table 2). Clinical stage data, available for only 79 of the patients, showed that the III (A&B) and IV (A&B) stages were found in 58.2% (46/79) of SCC. In addition, 36.96% (17/46) of patients at these III and IV stages were younger than 50 years of age (Table 3).

**Table 1:** Age of cervical cancer patients included in the study according to the histological diagnosis of the lesions (CIN3, ADC, SCC)

Age group (years)	Cervical intraepithelial neoplasia 3 (CIN3)	Adenocarcinoma (ADC)	Squamous cell carcinoma (SCC)	Total
< 39	3 (18.8%)	0	9 (8.4%)	12 (9.09%)
40–49	7 (43.8%)	2 (22.2%)	27 (25.2%)	36 (27.27%)
50–59	5 (31.2%)	5 (55.6%)	37 (34.6%)	47 (35.61%)
60–69	1 (6.2%)	1 (6.2%)	13 (12.1%)	15 (11.36%)
> 70	0	1 (11.1%)	21(19.6%)	22 (16.67%)
Total	16 (12.1%)	9 (6.8%)	107 (81.1%)	132 (100%)

**Table 2:** Tumor cell percentages according to histological diagnosis (CIN3, ADC, SCC)

	Tumor cell percentage			
	[5–40] n (%)	]40–50] n (%)	]50–80] n (%)	]80–100] n (%)
CIN3 (n = 16)	6 (37.5%)	4 (25%)	3 (18.75%)	3 (18.75%)
ADC (n = 8)	2 (25%)	1 (12.5%)	1 (12.5%)	4 (50%)
SCC (n = 107)	23 (23.5%)	24 (24.5%)	34 (34.7%)	17 (17.3%)

**Table 3:** Clinical stages of squamous cell carcinoma (SCC) by age group

Age group	Clinical stages of squamous cell carcinoma								
					n				
	IA	IB	IIA	IIB	IIIA	IIIB	IVA	IVB	Total
< 39	1	0	1	0	0	1	2	2	7
40–49	1	1	3	3	0	7	3	2	20
50–59	1	2	2	9	2	7	6	0	29
60–69	1	1	2	0	2	2	1	1	10
> 70	1	1	1	2	0	5	3	0	13
Total	5	5	9	14	4	22	15	5	79

### Overall prevalence of HPV

Of the 132 samples investigated in this study, at least one HPV was detected in 92.4% (122/132) even if the GAPDH gene was amplified in 67.42% (89/132) samples. However, only 64% of the different genotypes were detected with two methods used for our virological investigations. Nonetheless, 100% of the HPV genotypes detected with qPCR were also detected with conventional nested PCR (Additional file 1: Table S1). HPV prevalence was 77.8% (7/9), 93.6% (15/16) and 93.5% (100/107) among the ADC, CIN3 and SCC samples, respectively. Six different HPV genotypes (HPV16, 18, 33, 35, 45 and 58), all classified as high risk, were found in descending order: HPV16 75.4% (92/122); HPV18 18% (22/122); HPV58 10.7 (13/122); HPV45 9.8% (12/122); HPV33 4.1% (5/122) and HPV35 0.8% (1/122). Co-infections were identified in 28/122 cases (23%). HPV16 and HPV18 were both found in 82.8% (101/122) samples. The HPV16 genotype was the most frequent genotype, detected in 60% (15/25) and 77% (77/107) of ADC/CIN3 and SCC samples, respectively. The second most frequent high-risk genotype was HPV18 with percentages of 13.3% (2/15) to 28.6% (2/7) in CIN3 and ADC cases, respectively (Table 4) and in 15.0% (16/117) of SCCs. However, in 50% and 61.1% of cases, HPV18 was found in co-infection with HPV16 for ADC/CIN3 and SCC samples, respectively. The percentages of the other high-risk HPV genotypes in SCC varied between 1.1% and 11.7% (HPV35 and HPV58, respectively). Moreover, in 58.3% of cases, these minority genotypes were found in co-infection with either HPV16 or HPV18. Among CIN3 histological diagnostic 2/15 were found co-infected with HPV16 and HPV18 on an hand and HPV45 and HPV58 on an other hand. regarding the ADC histological diagnostic, 3/7 cases were co-infected. It was HPV16-HPV18; HPV16-HPV58 and HPV18-HPV45.

**Table 4:** HPV genotype detected according to histological diagnosis of the lesions (CIN3, ADC, SCC)

HPV genotype detected	Cervical intraepithelial neoplasia 3 (CIN3)	Adenocarcinoma (ADC)	Squamous cell carcinoma (SCC)
	n = 16 (%)	n = 9 (%)	n = 107 (%)
All genotypes	15 (93.8%)	7 (77.8%)	100 (93.5%)
HPV16	10 (66.7%)	5 (71.4%)	77 (77.0%)
HPV18	2 (13.3%)	2 (28.6%)	18 (18.0%)
HPV33	1 (6.7%)	0	4 (4.0%)
HPV35	0	0	1 (1.0%)
HPV45	2 (13.3%)	2 (28.6%)	8 (8.0%)
HPV58	1 (6.7%)	1 (14.3%)	11 (11.0%)

### HPV genotype prevalence in SCC according to age

All women with SCC were divided into five age groups (26–39; 40–49; 50–59; 60–69 and 70–87). Although the overall prevalence of at least one high-risk HPV was 87.5% (94/107), the prevalence differed according to age group. It varied from 86.7% (13/15) to 90.9% (20/22) for the 60–69 and 70–87 groups, respectively (Table 5). Moreover, regardless of the age group considered, HPV16 genotype was the most frequent genotype, varying from 61.7% (29/47) to 75% (27/36) for the 50–59 and 41–49 age groups, respectively. Except for HPV16, the only other HPV genotype found in several age groups was HPV18, even though it was found in co-infection with HPV16 in 61.1% (11/18) of the cases. The other less frequent genotypes (HPV33, 45 and 58) were not found in the first age group (26–39). In the other groups, they were detected either individually in 42.8% (9/21) of cases or in co-infection with HPV16 or 18 or with the another genotype. The HPV33 and 58 genotypes were found individually between two and four times, respectively, in all the age groups except for the 60–69 age group. Finally, no age group was statistically more infected than any other, either with HPV overall or with a particular HPV genotype (p = 0.8157).

**Table 5:** HPV genotype detected according to the age group of squamous cell carcinoma (SCC) patients

Age group (years)	26–39 years	40–49 years	50–59 years	60–69 years	70–87 years
n (%)	11/12 (91.7%)	33/36 (91.7%)	45/47 (95.7%)	13/15 (86.7%)	20/22 (90.9%)
HPV16	11	27	29	9	16
HPV18	2	5	9	3	3
HPV33	0	1	2	1	1
HPV35	0	1	0	0	0
HPV45	0	4	6	1	1
HPV58	0	2	6	1	4
HPV16, 18	2	4	5	1	1
HPV16, 45	0	1	1	1	1
HPV16, 58	0	1	2	1	3
HPV18, 58	0	0	1	0	0
HPV16, 18, 45	0	0	1	0	0

### HPV genotype prevalence in SCC according to clinical stage

Of the 107 SCCs in this study, histological stage (four groups, IA-IB, IIA-IIB, IIIA-IIIB and IVA-IVB) could be established for only 67 SCCs. At least one HPV was detected in 86.6% (58/67) of the cases, with the percentage of HPV detected ranging from 80.9% (17/21) to 93.3% (14/15) for groups IIA-IIB and IVA-IVB, respectively. In addition, in 66.2% (45/58) of the cases, a single HPV genotype was detected, albeit varying with age group: IA-IB was the group with the most co-infections, with only 28.5% of mono-infections; in the IIIA-IIIB group, 95% were mono-infections. The HPV16 genotype was found in all stage groups and was the most frequent regardless of stage group. Moreover, HPV16 was also the genotype most frequently found in mono-infections. In contrast, HPV18 was more often found in co-infection with HPV16 (7/11) than in mono-infection. Finally, the minority genotypes (HPV33, 45 and 58) were found in mono-infection only in groups IA-IB and IIA-IIB, but in 80% of co-infection cases with HPV16 in the other two groups IIIA-IIIB and IVA-IVB (Table 6).

**Table 6:** HPV genotype detected according to the clinical (FIGO) stage of SCC

	Clinical stage of SCC n (%)				
	IA/IB n = 8	IIA/IIB n = 21	IIIA/IIIB n = 23	IVA/IVB n = 15	N/A n = 38
All genotypes	7 (87.5%)	17 (80.9%)	20 (86.9%)	14 (93.3%)	34 (89.5%)
HPV16	2	10	15	8	20
HPV18	–	–	3	–	2
HPV33	1	–	–	–	3
HPV45	–	2	–	1	–
HPV58	–	2	–	–	2
HPV59	–	–	–	–	1
HPV16, 18	2	2	–	2	4
HPV16, 45	1	–	1	–	–
HPV16, 58	1	–	1	2	2
HPV18, 58	–	–	–	1	–
HPV16, 18, 45	–	1	–	–	–

## Discussion

Cervical cancer is the leading cause of cancer-related death among women in sub-Saharan Africa (SSA) but it can be prevented by vaccination. But, due to financial and logistical obstacles, their implementation is difficult in SSA. Although current vaccines can only prevent up to 9 different genotypes, it affords cross-protection between the genotypes targeted by the vaccine and non-target genotypes. However, the effectiveness of these campaigns also depends on a better understanding of the distribution of different HPV genotypes in women with high-grade lesions. Although many studies have been conducted over the past few decades in SSA countries, their comparison is difficult because of the lack of standardization in the methodologies used in pathology and virology. Although screening by molecular testing for HPV genomic DNA is more efficient than screening by cytology, histological confirmation of cases remains the cornerstone of clinical diagnosis. However, pathology laboratories are rare in low-income countries [15]. For example, to date in Gabon, there are only four laboratories all located in the capital city and the most recent of which became operational in 2020. However, two of these laboratories located at the *CHUA* and the *HIA OBO* have biobanks that can be used to assess the prevalence of different HPV genotypes in cervical cancer according to clinical stage. However, screening based on smears associated with cytological analysis has a low coverage rate due to the insufficient infrastructures and the lack of trained personne; HPV screening covers a greater proportion of the population, but is difficult to set up due to the need fo specific infrastructures unavailable on a large scale in Gabon. The global health situation due to COVID-19 has led to a large deployment of thermal cyclers in hospitals in the capital of Gabon as well as in the provinces [16]. Thus, the implementation of molecular screening now seems possible throughout the country.

To determine the prevalence of the different HPV genotypes associated with CIN3 + in the Gabonese population according to methodologies standardized with other French-speaking African countries belonging to the COFAC-Col consortium [7], they were screened on the FFPE samples. The proportions of HPV detected were lower than those reported in studies performed on smears or biopsies in the same Gabonese population [9–11]. There are two main reasons behind this absence of HPV detection in a small proportion of cancer biopsies [17, 18]. First, the DNA extracted from FFPE samples may be partially degraded, making HPV detection more difficult for technical reasons. Second,the target region of the PCR/qPCR used may have been deleted during the process of integration of the viral genome into that of the host. A metanalysis reported viral integration of HPV into the host genome in more than 80% of cervical cancers [19]. This proportion is similar to that obtained in a recent study performed in Gabon, in which the viral genome was found integrated in 85.7% of cervical cancer cases [20].

The diversity of HPV genotypes detected in this study is lower than that obtained in other studies conducted in other SSA countries. Indeed, only six genotypes were detected: HPV16, 18, 33, 35, 45 and 58. Previous studies conducted in Gabon reported between 11 and 24 different genotypes including the six high-risk genotypes in this study [9–11]. However, these studies were based on total DNA extracted from either cervical smears or non-paraffin biopsies, in contrast to this study where only high-grade tumor tissues were specifically collected from paraffin slides. This preselection decreases the probability of finding HPV genotypes not associated with cancer or high-grade lesions. For example, high-grade CIN is primarily caused by a single HPV type [21]. The diversity observed in Gabon is also lower than that observed in Senegal (HPV16, 18, 31, 33, 35, 39, 45, 56 and 58) and Cameroon (HPV16, 18, 33, 35, 39, 45, 51, 56 and 82) in FFPE samples, where nine genotypes were detected [22, 23]. Although diversity varies among SSA countries, the two main genotypes are still HPV16 and HPV18, with more than 75% of cases in Gabon. Finally, the clinical data for cervical cancer in our study are consistent with previous studies conducted in Africa, and particularly in SSA [24, 25]. Cervical cancer in Gabon mainly affects young women under 50 years of age and a large number of cervical cancer cases is diagnosed at a late stage, with 87.3% of patients at stage II or higher.

## Conclusion

This study was part of a transversal initiative between several French-speaking countries to standardize methods to obtain results generalizable across the SSA region. Our results confirm the high prevalence of the HPV16 and 18 genotypes among high-grade lesions in the Gabonese population. Among the six genotypes detected, five are targeted by the HPV 9-valent vaccine. This result highlights the need for a national strategy for the early screening of precancerous lesions coupled with a broad national vaccination program targeting non-sexually active women to significantly reduce the long-term cancer burden.

## Supplementary Information

**Additional file1**.** Table S1**. HPV genotype detected by histological stage according to molecular method

## Data Availability

All data are reported in the manuscript.

## Abbreviations

HPV: Human papillomavirus
CIN 3: Cervical intraepithelial neoplasia 3
ADC: Adenocarcinoma
SCC: Squamous cell carcinoma
FFPE: Formalin-fixed paraffin-embedded
