# Supplementary material for: HPV genotypes in high-grade cervical lesions and invasive cervical carcinoma detected in Gabonese women
Source: Infect Agent Cancer. 2023 Mar 8;18:16. doi: 10.1186/s13027-023-00493-z (PMC9994388; doi:10.1186/s13027-023-00493-z)
Supplement: Supplementary file 1 — Additional file1. Table S1. HPV genotype detected by histological stage according to molecular method [file 13027_2023_493_MOESM1_ESM.docx]

**Table S1: HPV genotype detected by histological stage according to molecular method**

|  | HPV genotype | Real-time PCR | Conventional nested PCR + Sanger sequencing |
| --- | --- | --- | --- |
| ADC | HPV16 | 4 | 5 |
|  | HPV18 | 2 | 2 |
|  | HPV45 | 2 | 2 |
|  | HPV58 | 1 | 1 |
| CIN3 | HPV16 | 4 | 10 |
|  | HPV18 | 1 | 2 |
|  | HPV33 | 1 | 1 |
|  | HPV45 | 2 | 2 |
|  | HPV58 | 1 | 1 |
| SCC | HPV16 | 52 | 86 |
|  | HPV18 | 10 | 18 |
|  | HPV33 | 2 | 4 |
|  | HPV35 | 0 | 1 |
|  | HPV45 | 5 | 8 |
|  | HPV58 | 11 | 11 |
